# A Pool Drowning Detection Model Based on Improved YOLO

**DOI:** 10.3390/s25175552

**Published:** 2025-09-05

**Authors:** Wenhui Zhang, Lu Chen, Jianchun Shi

**Affiliations:** 1 School of Computer Science and Information Security, Guilin University of Electronic Technology, Guilin 541004, China; 23032304041@mails.guet.edu.cn; 2 Jiangsu Zhaoming Information Technology Co., Ltd., Nantong 213000, China; 13961495188@139.com

**Keywords:** drowning detection, YOLO11, lightweight, attention, feature fusion

## Abstract

**Highlights:**

**What are the main findings?**

- The proposed YOLO11-LiB achieves a high drowning class mean average precision (DmAP50) of 94.1% while being extremely lightweight (2.02 M parameters, 4.25 MB size).
- Key innovations include the LGCBlock for efficient downsampling, the C2PSAiSCSA module for enhanced spatial–channel feature attention, and the BiFF-Net for improved multi-scale feature fusion.

**What is the implication of the main finding?**

- Addresses critical limitations in real-time drowning detection: poor edge deployment efficiency, robustness in complex water environments, and multi-scale object challenges.
- Provides a high-performance, computationally efficient solution enabling practical real-time surveillance in swimming pool scenarios.

**Abstract:**

Drowning constitutes the leading cause of injury-related fatalities among adolescents. In swimming pool environments, traditional manual surveillance exhibits limitations, while existing technologies suffer from poor adaptability of wearable devices. Vision models based on YOLO still face challenges in edge deployment efficiency, robustness in complex water conditions, and multi-scale object detection. To address these issues, we propose YOLO11-LiB, a drowning object detection model based on YOLO11n, featuring three key enhancements. First, we design the Lightweight Feature Extraction Module (LGCBlock), which integrates the Lightweight Attention Encoding Block (LAE) and effectively combines Ghost Convolution (GhostConv) with dynamic convolution (DynamicConv). This optimizes the downsampling structure and the C3k2 module in the YOLO11n backbone network, significantly reducing model parameters and computational complexity. Second, we introduce the Cross-Channel Position-aware Spatial Attention Inverted Residual with Spatial–Channel Separate Attention module (C2PSAiSCSA) into the backbone. This module embeds the Spatial–Channel Separate Attention (SCSA) mechanism within the Inverted Residual Mobile Block (iRMB) framework, enabling more comprehensive and efficient feature extraction. Finally, we redesign the neck structure as the Bidirectional Feature Fusion Network (BiFF-Net), which integrates the Bidirectional Feature Pyramid Network (BiFPN) and Frequency-Aware Feature Fusion (FreqFusion). The enhanced YOLO11-LiB model was validated against mainstream algorithms through comparative experiments, and ablation studies were conducted. Experimental results demonstrate that YOLO11-LiB achieves a drowning class mean average precision (DmAP50) of 94.1%, with merely 2.02 M parameters and a model size of 4.25 MB. This represents an effective balance between accuracy and efficiency, providing a high-performance solution for real-time drowning detection in swimming pool scenarios.

## 1. Introduction

Drowning constitutes a major global public health crisis that demands urgent attention, with its severity being impossible to overlook. According to the World Health Organization (WHO), the number of global drowning deaths reached a staggering 300,000 in 2021—equivalent to over 34 fatalities per hour [1]. Focusing on China, data from the China Youth Drowning Prevention Big Data Report 2022 [2] shows that drowning accounts for 33% of all injury-related deaths among Chinese adolescents. The high incidence of drowning incidents in 2022 further emphasizes the urgency of strengthening prevention and control measures.

Statistics indicate that China currently has a total of 39,700 swimming venues, primarily categorized into outdoor pools, indoor pools, and natural swimming sites. Specifically, outdoor pools (20,600 venues, accounting for 51.95%) and indoor pools (18,200 venues, accounting for 45.68%) together make up 97.63% of the total, while natural swimming sites are far less common (only 940 venues, comprising a mere 2.37%) [3].

As the most prevalent types of swimming facilities, indoor and outdoor pools pose particularly acute drowning risks. Drowning is characterized by its quiet and rapid progression, and this process is easily obscured by complex environmental factors—such as water surface glare and crowd movements—greatly undermining the effectiveness of manual surveillance [4]. Conversely, the inherent physical characteristics of these venues (fixed boundaries, clear water quality, and relatively controllable lighting) provide ideal, engineering-friendly conditions for deploying automated detection systems based on AI vision or sensor technology [5,6].

Therefore, researching drowning detection technology specifically for indoor and outdoor swimming pools—core scenarios marked by high drowning risks and strong technical feasibility—is of critical significance. It helps address blind spots in manual supervision, shorten rescue response times, and enhance the overall safety prevention and control capabilities of swimming facilities.

In recent years, drowning detection technologies have primarily developed along two technical pathways:

The first category relies on wearable sensing devices. These technologies collect physiological and behavioral data (e.g., swimmers’ heart rate, blood oxygen levels, water pressure, and movement status) to enable real-time behavior monitoring and emergency response [7]. However, this approach mandates that users wear the devices, which not only may impair the swimming experience but also presents practical challenges—such as inconvenience in use and poor adaptability for infants and young children—hindering its large-scale application [8].

The second category adopts vision-based deep learning methods, which use cameras to capture image data and leverage object detection algorithms to analyze swimmers’ behaviors [9]. With the rapid iteration of object detection techniques, single-stage detection models (represented by the YOLO series) have gradually become the mainstream choice for drowning detection. This is attributed to their advantages of strong real-time performance, low deployment costs, and excellent scene generalization capabilities [10]. Nevertheless, existing solutions still have room for optimization in several key dimensions:

From an engineering implementation perspective: Even lightweight models like YOLO11n impose significant burdens on the storage and computational resources of edge devices, due to their parameter scale, model size, and computational overhead. This makes them difficult to adapt to resource-constrained scenarios (e.g., pool-embedded terminals) [11]. From a detection performance perspective: in complex aquatic environments, factors such as water surface glare interference and target occlusion can reduce the model’s focus on subtle drowning posture features, thereby weakening the robustness of recognition [12]. From a multi-scale target adaptation perspective: drowning scenarios often involve targets of varying scales (e.g., nearby swimmers vs. distant drowning individuals). Current models’ multi-scale feature fusion strategies are still inadequate, frequently leading to missed detection of small targets or insufficient feature extraction for large targets [13,14].

To address the aforementioned challenges, this paper proposes the YOLO11-LiB model, which is designed to enhance existing object detection algorithms for improved drowning detection performance and provide a more effective solution to the identified problems. Specifically, the model achieves three key optimizations:

It reduces computational complexity through lightweight dynamic convolution and optimized downsampling operations, meeting the requirements for real-time early warning; it incorporates a residual-structured separation attention mechanism to adaptively adjust feature weights, suppress irrelevant interference, enhance sensitivity to drowning victim features, and reduce false positives and false negatives caused by complex backgrounds; and it employs advanced multi-scale feature fusion to capture detailed information of targets at different scales, mitigating the degradation of detection accuracy caused by distance variations or pose changes of drowning individuals, and thus improving detection performance in complex scenarios.

The main contributions of this study are summarized as follows:

1. Lightweight Feature Extraction Design: We propose the Lightweight Feature Extraction Module (LGCBlock), which integrates the Lightweight Attention Encoding Block (LAE) and effectively combines Ghost Convolution (GhostConv) with Dynamic Convolution (DynamicConv). This module optimizes the downsampling structure and the C3k2 module in the YOLO11n backbone network, significantly reducing model parameters and computational complexity while preserving the integrity of key features.
2. Enhanced Attention Mechanism for Robust Feature Extraction: We introduce the Cross-Channel Position-aware Spatial Attention Inverted Residual with Spatial-Channel Separate Attention module (C2PSAiSCSA) into the backbone network. This module embeds the Spatial–Channel Separate Attention (SCSA) mechanism within the Inverted Residual Mobile Block (iRMB) framework, enabling more comprehensive and efficient feature extraction. It enhances the perception of subtle drowning postures and effectively suppresses interference from water surface glare and crowd occlusion.
3. Multi-scale Feature Fusion Architecture: We redesign the neck structure as the Bidirectional Feature Fusion Network (BiFF-Net), which integrates the Bidirectional Feature Pyramid Network (BiFPN) and Frequency-Aware Feature Fusion (FreqFusion). This design optimizes the semantic fusion of multi-scale features through a bidirectional feedback mechanism of dynamic weighting and frequency-domain enhancement, significantly improving detection accuracy for multi-scale targets—particularly small drowning victims.
4. Comprehensive Performance Evaluation: We establish a multi-dimensional evaluation system and validate the proposed YOLO11-LiB model through comparative experiments with state-of-the-art algorithms and ablation studies. The results demonstrate that our model achieves a drowning class mean average precision (DmAP50) of 94.% with only 2.02 M parameters and a model size of 4.25 MB, effectively balancing accuracy and efficiency for real-time drowning detection in swimming pool scenarios.

## 2. Existing Studies

### 2.1. Sensor-Based Drowning Detection Technologies

Early research on drowning detection primarily relied on wearable devices or environmental sensors to collect physiological and motion parameters. For example, Palaniappan et al. [15] developed a wristband-type wireless sensor system: it triggers alerts by comparing the moving average of accelerometer data with preset thresholds, while simultaneously activating an airbag inflation device to enable active rescue. However, this method depends on single-dimensional motion data, making it prone to false alarms when swimmers perform intense movements.

Jebamalar et al. [16] further integrated heart rate oximeters, triaxial accelerometers, and water pressure sensors with Global Positioning System (GPS) positioning technology to achieve multi-parameter fusion detection. Similarly, Gumaei et al. [17] demonstrated the effectiveness of hybrid deep learning models in fusing multimodal body sensing data for complex activity recognition, which provides a valuable reference for multi-sensor drowning detection systems. Nevertheless, in scenarios involving turbid water or multipath interference, sensor signals are still vulnerable to attenuation, affecting detection reliability.

Basthikodi et al. [18] designed a monitoring system based on ESP8266 and Arduino boards, which incorporates environmental parameters such as body temperature and humidity for comprehensive monitoring. However, the system’s wired connection design severely restricts its practical deployment scenarios, limiting its applicability in large-scale swimming venues.

As pointed out by Ganesamoorthy et al. [19], traditional video surveillance methods based on the HSV color space experience a significant decline in detection rates when targets are not fully submerged or in turbid water environments. Although sensor-based approaches can make up for the blind spots of visual detection, they have inherent drawbacks—such as poor wearing comfort and high risk of detachment—making them particularly unsuitable for infants and young children [20].

### 2.2. Deep Learning-Based Visual Detection Technologies

With the iterative advancement of object detection algorithms, vision-based solutions have gradually become a research focus due to their non-contact advantage. Chen et al. [21] proposed a lightweight network based on forward-looking sonar images, which integrates Bottleneck Transformer and feature pyramids to address the limited imaging range of underwater optical systems. Other sensing modalities like mmWave radar have also been explored for robust perception; for instance, Kosuge et al. [22] developed a real-time multiclass recognition system based on mmWave imaging radar, showcasing its potential for all-weather applications. However, the low resolution of sonar images or radar point clouds makes it difficult to distinguish between drowning postures and normal diving postures, leading to potential misjudgments.

YOLO series algorithms have been widely applied in drowning detection, thanks to their favorable balance between real-time performance and detection accuracy. Yang et al. [23] enhanced the YOLOv5s model by innovatively introducing an ICA module (integrating Coordinate Attention (CA) and Sigmoid Linear Unit (SiLU) activation functions) and a BiFPN feature fusion structure. The work by Sun et al. [24] on arbitrary-oriented object detection in SAR images also exemplifies the trend of designing specialized network architectures (like their BiFA module) to address challenges in specific visual domains. Their model achieved a detection accuracy of 98.1% in indoor swimming pool scenarios, but it still showed limitations in complex environments with multi-target occlusion. Jiang et al. [25] proposed the Swimming-YOLO model, which dynamically adjusts the position of sampling points through deformable convolutions and embeds a deformable attention mechanism to enhance the extraction of detailed features. Although this model demonstrates better comprehensive performance than the traditional YOLOv8 on multi-scene datasets, its large parameter size poses challenges for deployment on embedded devices with limited resources.

For complex outdoor water environments, Liu et al. [26] proposed the lightweight YOLOv8-REH model. They designed a C2f-RVB-ELA feature extraction module and an ELA-HSFPN fusion structure, and combined them with the Powerful-IoUv2 loss function and layer-adaptive pruning technology. This approach compressed the model size to 1.8 MB and increased the frame rate by 22.9 FPS. However, the model’s detection accuracy for small targets under backlight or wave interference conditions still needs further optimization. He et al. [20] compared the performance of Faster R-CNN and YOLOv5 variants, and found that YOLOv5s achieved the best results in infant drowning detection: it reached 89% mAP at a frame rate of 75 FPS, which was significantly superior to Faster R-CNN’s 6 FPS. This confirms the significant real-time advantage of single-stage detection algorithms in drowning detection tasks.

Gao et al. [27] built a pose analysis system based on the YOLOv8-POSE model, which identifies drowning behavior by detecting abnormal changes in human key points. To better model the temporal dynamics of such postural changes, techniques from other domains like Spatio-Temporal Graph Convolutional Networks (ST-GCNs) [28] could be insightful, as they are specifically designed to capture spatial and temporal dependencies in graph-structured data like human skeletons. However, the system relies on continuous pose sequences for judgment, resulting in a response delay of up to 1.2 s in sudden drowning scenarios—this delay may miss the optimal rescue window. Jebamalar et al. [16] noted that current models still lack sufficient robustness in handling multi-scale targets (e.g., coexisting adults and children) and dynamic backgrounds (e.g., water flow fluctuations). Nevertheless, the iterative upgrades of YOLO series algorithms (such as the C3k2 structure and improved loss functions in YOLO11) provide a solid technical foundation for addressing these challenges.

These studies highlight three core advantages of YOLO series algorithms in drowning detection:

- Real-time performance: The single-stage architecture supports high frame rates, meeting the requirement of rapid response in drowning emergencies;
- Environmental adaptability: The integration of attention mechanisms and feature fusion structures optimizes performance in complex scenarios;
- Lightweight potential: Compact model designs enable deployment on edge devices.

However, existing improved models based on YOLOv5/v8 still have limitations: low recall rates in multi-target occlusion scenarios, high miss rates for small targets under strong outdoor light, and insufficient modeling of the dynamic evolution process of drowning postures.

Considering these factors, this study selects YOLO11n as the base model for further enhancement. Its upgraded backbone network and detection head design have inherent potential to address the aforementioned challenges. By introducing dynamic attention mechanisms and multi-scale feature alignment modules, we aim to further improve the detection accuracy and robustness of the model in complex water environments.

## 3. Method

**Figure 1:**
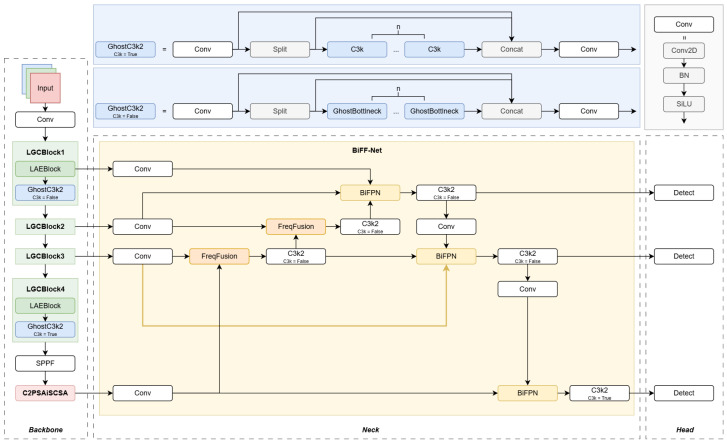
YOLO11-LiB network structure, composed of backbone, neck and head.

The overall architecture of YOLO11-LiB is illustrated in Figure 1. Based on the Ultralytics YOLO11n framework, our model introduces targeted enhancements for drowning detection in complex aquatic environments, adopting a multi-scale output structure. Key modifications include:

In the backbone, traditional downsampling is replaced with the lightweight LAE module. Combined with the GhostC3k2 block, this enables efficient feature extraction. An enhanced Inverted Residual module with Spatial–Channel Separate Attention (C2PSAiSCSA) is added at the backbone’s end to boost focus on fine-grained details of small targets.

Within the neck, feature channels from different scales are first standardized. Feature representation is then refined through FreqFusion and multiple C3k2 blocks. Contextual interaction is enhanced using the bidirectionally weighted BiFPN, followed by cross-scale downsampling via strided convolutions. The fused features are finally fed into the detection head for prediction.

This architecture significantly improves detection accuracy and inference efficiency through multi-level attention and frequency domain fusion, meeting the real-time and robustness requirements for drowning detection.

### 3.1. LGCBlock

**Figure 2:**
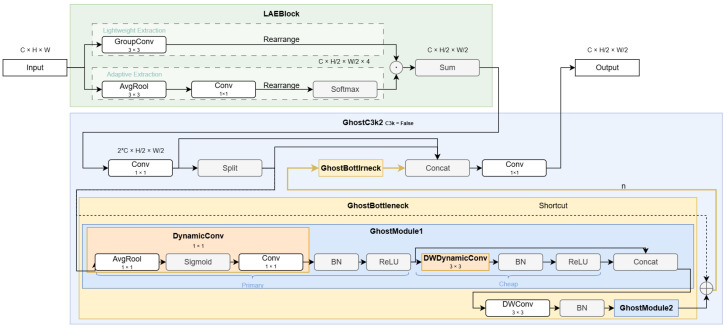
The structure of LGCBlock comprises LAE and GhostC3k2.

To enhance edge inference efficiency, we propose the LGCBlock, integrating a Lightweight Attention-based Extraction (LAE) module and a GhostC3k2 module (Figure 2), addressing computational redundancy and information loss in the original YOLO11n backbone.

To address downsampling optimization, this paper introduces the Lightweight Attention-based Extraction (LAE) module (Yu et al., 2024, LSM-YOLO [29]), which achieves efficient feature compression via dual-branch cooperation (Figure 2). The attention branch employs 3 × 3 average pooling and 1 × 1 convolution to generate spatial weights for 2 × 2 regions. The downsampling branch uses grouped convolution for 2× reduction. Features are reshaped to [B,C,H/2,W/2,4], fused via softmax-normalized weights and weighted summation, reducing computation to 12.5% of standard convolution while preserving critical information.

To reduce C3k2’s complexity, we design a GhostModule using dynamic convolution. Its primary path applies 1 × 1 DynamicConv (with a routing network combining four experts) for channel compression. The secondary path uses 3 × 3 depthwise separable dynamic convolution. Given output channels $C_{\mathrm{out}}$, the primary branch produces $m=\left\lceil \frac{C_{\mathrm{out}}}{r}\right\rceil$ channels (r=2), and the secondary branch $n=C_{\mathrm{out}}-m$, halving computation. This extends to a GhostBottleneck with channel expansion, depthwise convolution (for stride > 1), and channel compression, integrated with adaptive shortcuts and DropPath.

Integrated into the backbone, LAE replaces downsampling layers, while GhostC3k2 modifies C3k2 by dynamically using GhostBottlenecks (c3k = False) or retaining C3k (c3k = True), preserving residual connections.This synergy significantly reduces the computational load of the LGCBlock while maintaining accuracy, enabling efficient edge deployment for drowning detection.

### 3.2. C2PSAiSCSA

The original C2PSA module in YOLO11n lacks effective spatial modeling and dynamic adaptability, limiting its perception of subtle drowning movements in complex aquatic environments. To address this, we design the C2PSAiSCSA module by integrating an inverted Residual Mobile Block (iRMB) for local context and a Spatial–Channel Separate Attention (SCSA) mechanism for long-range dependencies.

**Figure 3:**
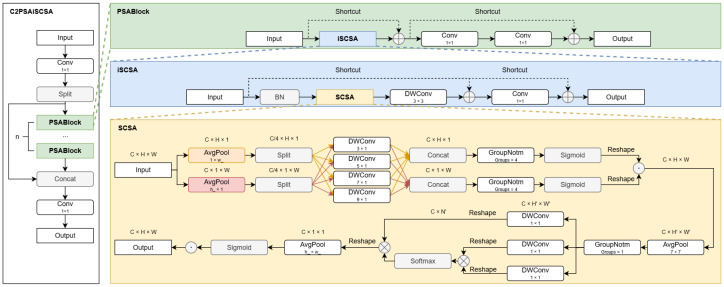
The structure of C2PSAiSCSA.

As illustrated in Figure 3, the core innovation is the iSCSA unit, which embeds SCSA within iRMB’s residual framework. Input features are first normalized and processed by SCSA to generate spatial-channel attention maps. Depthwise convolution then models local context and performs channel recalibration, followed by a projection layer and residual connection.

This iSCSA unit forms the PSABlock, which replaces the attention branch in the original C2PSA. The overall structure retains the dual-path design: one path preserves features via a skip connection, while the other undergoes deep enhancement through stacked PSABlocks. Each PSABlock contains an iSCSA module and a Feedforward Network (FFN). The iSCSA module employs an inverted residual structure: it expands channels, performs spatial modeling with depthwise separable convolution (enhanced by the integrated SCSA), and then compresses channels. The embedded SCSA uses multi-scale depthwise convolution for spatial attention and multi-head attention for channel selection, guiding the model to focus on critical regions.

Finally, features from the enhanced path are concatenated with the skip-connected features and integrated via a 1×1 convolution. This design significantly improves spatial perception and feature selectivity for small drowning targets under lightweight constraints, enhancing robustness and accuracy in complex backgrounds without substantially increasing computational cost.

### 3.3. BiFF-Net

**Figure 4:**
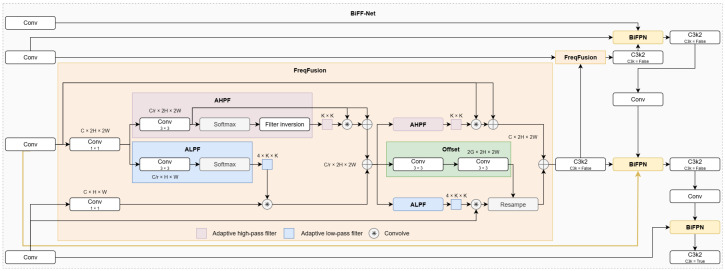
The structure of BiFF-Net.

The original YOLO11n neck structure (FPN + PAN) employs a static fusion mechanism, struggling to balance semantic and detail features for small, low-contrast drowning targets amidst complex aquatic interference. To overcome this, we propose the Bidirectional Feature Fusion Network (BiFF-Net), integrating BiFPN and FreqFusion for dynamic, robust multi-scale fusion (Figure 4).

The BiFPN module introduces learnable weights, normalized via a Swish activation, to dynamically modulate the contribution of input features from different scales. This adaptive weighting enhances critical features (e.g., target edges) while suppressing redundancies (e.g., water ripple noise), significantly improving fusion flexibility and robustness.

The FreqFusion module operates in the frequency domain to compensate for detail loss. It generates content-aware adaptive low-pass (ALPF) and high-pass (AHPF) filters. The low-pass branch upsamples and smooths deeper features using CARAFE with ALPF to emphasize semantic contours. The high-pass branch applies AHPF to shallower features to accentuate high-frequency details (e.g., fine edges, textures). The outputs are summed, producing features rich in both global semantics and local details.

BiFF-Net establishes a synergistic “Frequency Enhancement → Dynamic Weighting” workflow. Multi-scale features are first enhanced by FreqFusion to repair degradation (e.g., blurred contours). BiFPN then dynamically fuses these enhanced features. This mechanism enables dynamic cross-level interaction, effectively suppressing background interference and compensating for detail loss, thereby significantly improving perception and localization accuracy for challenging drowning targets while maintaining efficient inference.

## 4. Experiments

### 4.1. Dataset

This study constructs a dedicated drowning detection dataset covering both indoor and outdoor swimming pool environments, comprising a total of 2000 original images. Through extensive data augmentation techniques (including rotation, scaling, brightness adjustment, saturation variation, hue shift, zoom, blur, noise injection, and mosaic augmentation), the dataset was expanded to a total of 4800 images for model training. Data was sourced from the public dataset platform Roboflow (https://roboflow.com/) and open internet resources (including videos and images), encompassing both above-water and underwater perspectives.

To maximize diversity and real-world applicability, the dataset was meticulously curated across multiple dimensions:

- Scenario Diversity: Includes indoor heated pools and outdoor open-air pools, with different lane designs and poolside environments.
- Environmental Diversity: Covers various lighting conditions (e.g., bright midday sunlight, low evening light, artificial nighttime lighting, and light refraction underwater), different weather conditions (sunny, cloudy), and varying water clarity.
- Subject Diversity: Encompasses individuals of different age groups (children, adults, the elderly), body types, skin tones, and those wearing different swimwear (e.g., dark, light, bikinis, swimsuits).
- Behavioral Diversity: Data includes not only swimming and drowning subjects but also rich background activities such as walking poolside, diving, and playing in the water, enhancing the model’s ability to discern targets in complex scenes.
- Technical Diversity: Data was obtained from various capture devices (e.g., surveillance cameras, action cameras, smartphones), introducing variations in resolution, viewing angles (overhead, eye-level, oblique), and aspect ratios.

The dataset was partitioned into training, validation, and test sets in a 7:2:1 ratio. All images were meticulously annotated in YOLO format using Roboflow tools. The annotation categories are defined as follows:

- Swimming: Represents normal and controlled swimming postures. Characteristics typically include a streamlined, near-horizontal body position; rhythmic and coordinated limb movements (e.g., regular arm strokes and leg kicks); the head is often raised for breathing or submerged rhythmically; and movement direction is purposeful.
- Drowning: Represents active or imminent drowning behavior, characterized fundamentally by a loss of voluntary motor control. Specific manifestations include a vertical or upright, tilted body position with an inability to swim effectively; arms may extend laterally or slap the water involuntarily (Instinctive Drowning Response) without providing propulsion; the head may be submerged for extended periods or tilted back with the mouth seeking air, often with a glassy-eyed stare; leg movement is minimal or absent, often leading to a gradual submersion after struggle.

To mitigate overfitting risks due to the extended training of 500 epochs, the aforementioned data augmentation strategies were rigorously applied. Furthermore, the training process was closely monitored. The loss curves for both training and validation sets demonstrated a convergent trend without significant divergence, indicating that overfitting was effectively controlled. The loss curves were analyzed in detail in the subsequent chapters.

**Figure 5:**
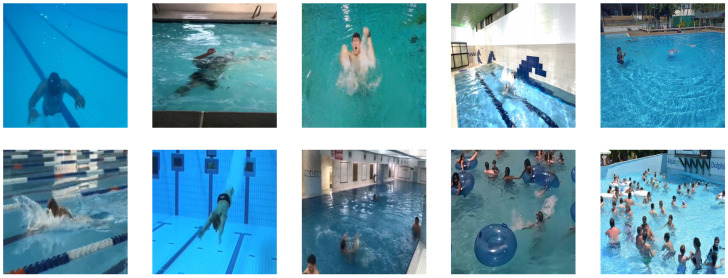
Examples of the dataset include scenarios with mixed samples of different perspectives, different environments, multi-targets, and small targets.

Figure 5 showcases representative samples from the dataset, providing a visual comparison of the two behavioral modes. This dataset has been publicly released on the Roboflow platform.

### 4.2. Evaluation Metrics

This study employs multiple evaluation metrics to comprehensively assess model performance, including Drowning Precision (DP), Drowning Recall (DR), Drowning mean Average Precision (DmAP50), Swimming mean Average Precision (SmAP50), Frame Rate (FPS), Floating Point Operations (FLOPs, in G), Number of Parameters (P/M, in millions), and model size (Size/M, in MB).

Specifically, Drowning Precision measures the accuracy of predictions for the drowning class, representing the proportion of true drowning instances among samples predicted as drowning. Drowning Recall quantifies the detection capability for drowning instances, indicating the proportion of actual drowning cases correctly identified by the model.

DmAP50 and SmAP50 denote the mean average precision for drowning and swimming classes respectively at an Intersection over Union (IoU) threshold of 0.5, reflecting the comprehensive performance of detection precision and recall capability for both target categories. The Frame Rate (FPS) evaluates the model’s real-time inference performance, demonstrating its capability for instantaneous detection.

FLOPs represent the computational complexity of the model, while the Number of Parameters and model size indicate the model’s scale and storage requirements, respectively, assessing its deployment feasibility on resource-constrained devices. Collectively, these metrics comprehensively characterize the proposed model’s overall performance across two dimensions: detection effectiveness and model efficiency.

### 4.3. Experimental Setup and Convergence Analysis

All experiments in this study were conducted within the PyCharm 2024.3.3 (Professional Edition) integrated development environment. The hardware configuration includes an NVIDIA GeForce RTX 3090 GPU (manufactured by NVIDIA Corporation, Santa Clara, CA, USA) and an Intel(R) Xeon(R) Platinum 8362 CPU (manufactured by Intel Corporation, Santa Clara, CA, USA) (15 cores, 2.80 GHz base frequency), running on the Windows 11 operating system. The software environment utilizes Python 3.10, with PyTorch 2.0.0 as the deep learning framework, supported by CUDA 11.8 acceleration.

Experimental hyperparameters were carefully tuned for the drowning detection task, with key parameters detailed in Table 1. The configuration follows established practices for YOLO-series models while incorporating adjustments specific to our application scenario. This setup enabled both our proposed model and the baseline models to achieve optimal performance.

**Figure 6:**
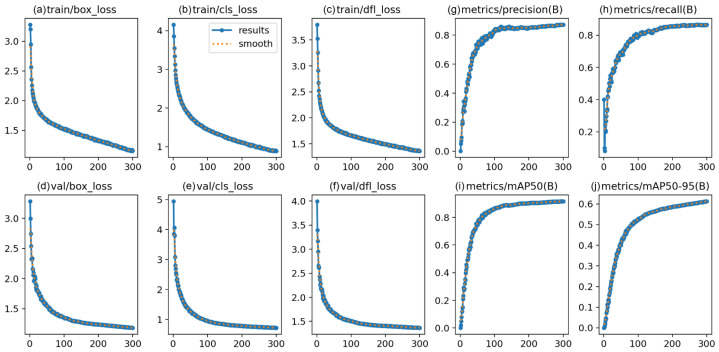
Training and validation metrics over 300 epochs. The top-left panels (**a**–**c**) show the training losses (box, cls, dfl). The bottom-left panels (**d**–**f**) show the corresponding validation losses. The right panels (**g**–**j**) illustrate the performance metrics on the validation set, including precision, recall, mAP@50, and mAP@50–95.

The training process and convergence behavior were meticulously analyzed. Extensive data augmentation was employed to mitigate overfitting risks associated with the limited dataset size. Figure 6 presents the trajectories of the training and validation losses alongside key performance metrics over the 300-epoch training course.

Analysis of the curves reveals a rapid decline in both training and validation losses during the initial 50 epochs, indicating efficient feature learning. The rate of decrease gradually slowed, with losses stabilizing after approximately 200 epochs, suggesting model convergence. Crucially, the validation loss closely tracked the training loss throughout the entire process without significant divergence, demonstrating strong generalization and a lack of severe overfitting. This is further supported by the performance metrics on the validation set. The mAP@50 exhibited a swift increase from near zero, surpassed 0.8 around epoch 100, and eventually plateaued at a high value of 0.941 by epoch 300.

The learning rate scheduling strategy contributed to this stable convergence. The learning rate was gradually reduced from its initial value of 0.01 to a final value of 0.000133, allowing for precise parameter tuning in the later stages of training without introducing instability.

In conclusion, the combination of a carefully chosen experimental setup, extensive data augmentation, and a tailored training regimen resulted in a stable and effective learning process. The model converged reliably over 300 epochs, achieving high performance on the validation set without exhibiting signs of overfitting, thereby validating the chosen experimental parameters and overall approach.

### 4.4. Comparative Experiments

To validate the effectiveness of the proposed YOLO11n-based improved model, this study selected multiple mainstream YOLO series versions, advanced object detection models, and representative non-YOLO series models for comparative experiments under identical datasets and experimental conditions. The results are presented in Table 2. To ensure the robustness of the conclusions, all metrics are reported as the mean with a 95% confidence interval (CI) from five independent runs (*n* = 5), and statistical significance was assessed using paired *t*-tests (α = 0.05).

The improved model demonstrates outstanding performance in drowning detection: Drowning Precision (DP) reaches 88.4% (88.4 ± 0.4%), and Drowning Recall (DR) reaches 89.7% (89.7 ± 0.5%), significantly outperforming the lightweight models YOLO11n (DP: 85.7 ± 0.5%, DR: 88.8 ± 0.6%) and YOLO12n (DP: 80.8 ± 0.8%, DR: 83.3 ± 0.7%) (*p* < 0.05) while matching the performance of the medium-sized models YOLO11s and YOLO11m, and surpassing traditional one-stage detectors like RetinaNet (DP: 84.9 ± 0.6%, *p* < 0.05). The drowning mean Average Precision (DmAP50) was 94.1% (94.1 ± 0.3%), which is statistically superior to most comparison models, including YOLO11n (91.8 ± 0.4%, *p* < 0.01), YOLOv5 (92.9 ± 0.3%, *p* < 0.05), and Faster R-CNN (92.8 ± 0.5%, *p* < 0.05). It demonstrates highly competitive performance against the state-of-the-art ViTDet (94.5 ± 0.6%), with the difference being not statistically significant (*p* > 0.05), which significantly enhances the detection accuracy for critical targets. For swimming detection, the improved model achieves 85.6% (85.6 ± 0.4%) SmAP50, outperforming most baseline models (e.g., vs. YOLO11n: 82.4 ± 0.5%, *p* < 0.01) and demonstrating robust multi-class recognition capability.

Although the inference speed (FPS) of the improved model is approximately 80.5 (80.5 ± 2.5) frames/second, lower than some models like YOLO11m and YOLO11l, it still meets the real-time requirements for drowning detection scenarios. In terms of model efficiency, the computational complexity (FLOPs) is only 6.2 G (6.2 ± 0.1 G), with approximately 2.02 million parameters and a model size of just 4.25MB, significantly lower than RTDERT-I, large-scale YOLO11 models, Faster R-CNN, ViTDet, etc., facilitating deployment in resource-constrained environments.

Minor declines in certain metrics (such as inference speed and detection accuracy for specific classes) primarily stem from the computational overhead introduced by enhanced feature representation and multi-scale fusion capabilities. Specifically, while multi-layer attention modules, frequency domain feature fusion, and weighted feature pyramid structures significantly enhance recognition of drowning targets in complex scenes, they also introduce additional computational burden, resulting in slightly reduced inference efficiency. Simultaneously, the deepened backbone network and detection head structures, designed to improve detection accuracy, further increase model complexity, leading to trade-offs in some lightweight metrics. Overall, this balance reflects a reasonable compromise between accuracy and efficiency, better aligning with the high precision requirements of practical drowning detection applications.

In summary, the proposed improved model effectively reduces model complexity and storage requirements while maintaining high detection precision and recall, achieving an optimal balance between detection performance and lightweight design. The statistical analysis confirms that the performance improvements are robust and significant, demonstrating broad applicability and promotion value for practical drowning detection applications.

### 4.5. Ablation Study

To systematically evaluate the impact of each proposed module on the drowning detection performance of YOLO11n, this paper conducts ablation studies: the lightweight module LGCBlock, attention-enhanced module C2PSAiSCSA, and feature fusion module BiFF-Net are incrementally integrated into the baseline model to investigate their individual contributions to detection performance and operational efficiency. The results, presented in Table 3, are reported as mean ±95% CI from five independent runs, with statistical significance (paired *t*-test, α = 0.05) calculated against the baseline (YOLO11n).

First, after introducing the lightweight downsampling feature extraction module (denoted as A), which integrates the attention-enhanced properties of LAE and the efficient convolution design of GhostC3k2, the model’s computational complexity and parameter count are significantly reduced (FLOPs: 5.9 ± 0.1 G vs. 6.3 ± 0.1 G; Params: 2.21 M vs. 2.58 M). This module effectively decreases FLOPs and model size, enhancing lightweight performance. However, due to a trade-off in feature representation capability, a slight decline in precision and recall for the drowning category is observed (DmAP50: 92.0 ± 0.4% vs. 91.8 ± 0.4%, *p* > 0.05), illustrating the inherent balance between lightweight design and detection performance.

Second, when independently incorporating the inverted residual separated attention module C2PSAiSCSA (denoted as B), its spatial–channel selective attention mechanism strengthens the model’s ability to capture critical features within drowning target regions. This results in a significant improvement in drowning category detection precision and mAP (DmAP50: 93.0 ± 0.3% vs. 91.8 ± 0.4%, *p* < 0.01). Simultaneously, while maintaining low computational complexity, this module effectively enhances the model’s capability to recognize small targets against complex backgrounds, achieving substantial performance gains.

Third, after independently integrating BiFF-Net (denoted as C), the optimized weighted fusion process for multi-scale features enriches the interaction and propagation of semantic information. This strategy not only further boosts detection recall (DR: 90.1 ± 0.6%) but also plays a crucial role in reducing computational resource consumption and model size, thereby enhancing the model’s practicality and deployment efficiency.

Finally, modules are progressively combined: The YOLO11n+A+B configuration introduces the attention mechanism on the lightweight foundation, achieving a balance between detection accuracy (DmAP50: 92.3 ± 0.4%) and model efficiency. The YOLO11n+A+B+C configuration leverages the synergistic effect of all three modules, yielding optimal performance in drowning category precision, recall, and mAP (DmAP50: 94.1 ± 0.3% vs. baseline 91.8 ± 0.4%, *p* < 0.001). Although inference speed exhibits minor fluctuations, it remains within application requirements, while computational complexity and parameter size are substantially reduced.

The ablation studies comprehensively validate the synergistic effects of lightweight design, attention mechanisms, and efficient feature fusion. The statistical analysis confirms the significant contribution of each module, demonstrating the targeted contributions of each module in enhancing performance and optimizing efficiency, ultimately achieving an ideal balance between accuracy and efficiency for the drowning detection task.

### 4.6. Visualization Result Analysis

**Figure 7:**
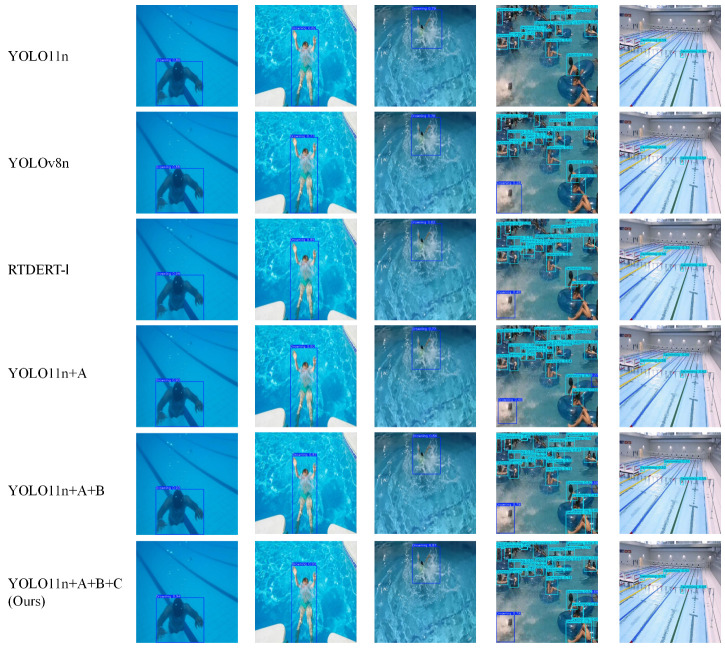
The detection results of different methods.

To intuitively demonstrate the advantages of the proposed algorithm in complex real-world scenarios, particularly its capability to address challenges such as water surface glare, crowd occlusion, and small object detection, this section provides a detailed qualitative analysis, as shown in Figure 7.

As shown in the second example column, under interference from complex bright backgrounds caused by strong water surface glare, the baseline model YOLO11n was able to detect the drowning person but with relatively low confidence. In contrast, our model—leveraging the powerful spatial–channel attention mechanism of the C2PSAiSCSA module—effectively suppresses glare-induced background noise and focuses on authentic target features. This results in more complete and accurate detections without false positives.

As illustrated in the third and fourth example columns, in scenarios with dense crowds and splash occlusion between targets, YOLO11n exhibits low confidence in detecting partially occluded targets due to its limited receptive field and insufficient feature extraction capability. In comparison, the C2PSAiSCSA module in our model enhances the ability to focus on key parts of the targets (such as heads and arms), effectively penetrating visual occlusions. It successfully localizes and identifies occluded drowning targets, significantly improving the model’s robustness.

As demonstrated in the fifth example column, for distant small-scale targets (with bounding boxes occupying less than 32 × 32 pixels), the detection confidence of all comparative models declines. Notably, YOLO11n and YOLOv8n show high missed-detection rates, while other models remain effective in identifying these targets. By bidirectionally integrating deep semantic information with shallow high-resolution features, the proposed BiFF-Net module supplies the detection head with rich, fine-grained details of small targets. As a result, our method achieves the highest and most stable detection performance for small objects, successfully validating the design objective of this module.

In summary, the visualization results strongly corroborate, from a qualitative perspective, the superior performance of the proposed model in addressing critical challenges in practical deployment, especially empowered by its core modules, which aligns with and reinforces the conclusions drawn from the previous quantitative analysis.

## 5. Discussion

This study enhances the YOLO11n model to address the specific challenges of drowning detection in complex aquatic environments. The discussion below clarifies the novelty of our contributions relative to prior YOLO-based improvements and explores the practical deployment considerations of the proposed model.

### 5.1. Novelty and Adaptation Relative to Prior YOLO Improvements

Our work stands on the shoulders of extensive research that has leveraged the YOLO architecture for drowning detection, as reviewed in Section 2. We consciously integrate several advanced concepts from the broader object detection domain while introducing targeted innovations to create a more effective and efficient solution.

The LGCBlock is a composite module designed for extreme lightweightness. Its LAE component is adapted from the work of Yu et al. [29], repurposing their efficient downsampling technique for our context. However, its integration with our novel GhostC3k2 module, which utilizes dynamic convolution to reduce computational complexity, represents a novel architectural choice not found in standard YOLO implementations or prior drowning detection models like [23,26]. This synergy directly addresses the common trade-off between speed and accuracy, a core limitation in deployment-focused studies such as [26].

The C2PSAiSCSA module introduces a significant innovation to enhance feature representation. While the idea of enhancing attention is prevalent (e.g., the ICA module in [23]), our approach is distinct. We novelly embed a Spatial–Channel Separate Attention (SCSA) mechanism within an inverted Residual Mobile Block (iRMB) framework. This creates a deeply integrated iSCSA unit that simultaneously captures local context and long-range dependencies more effectively than prior adaptations of coordinate or channel attention. This is particularly crucial for discerning subtle drowning postures amidst noisy water surfaces, a challenge highlighted by [16].

The BiFF-Net in the neck demonstrates a hybrid adaptation-and-innovation strategy. The use of BiFPN is an adaptation of a known technique for weighted feature fusion, similar to concepts in [23]. The novelty lies in its combination with our proposed FreqFusion module. This frequency-domain approach to feature enhancement, using adaptive filters to repair blurred contours and accentuate details, is a novel contribution to drowning detection. It directly tackles the problem of detail loss in low-contrast targets and wave interference, which limits the performance of models like [26].

In summary, our novelty does not solely lie in inventing entirely new operators but in the strategic integration and customized redesign of existing concepts into a cohesive architecture specifically optimized for the challenges of drowning detection, achieving a balance not present in prior art.

### 5.2. Deployment Considerations and Applicability

The design choices of YOLO11-LiB are intrinsically guided by practical deployment needs, namely handling multi-camera feeds under strict latency constraints.

Multi-Camera Support: The significant reduction in computational complexity and parameter count, primarily achieved through the LGCBlock, is the key to multi-camera deployment. A lighter model requires less computational resources per video stream, allowing a single edge computing device (e.g., an NVIDIA Jetson module) to process feeds from multiple cameras concurrently. This makes our model suitable for monitoring large pool areas or several zones within a natural water body without a linear increase in hardware cost.

Latency Constraints: The model’s architecture is engineered for high inference speed. The LAE module reduces computational overhead during downsampling, and the GhostC3k2 module streamlines feature extraction. These optimizations collectively ensure a high frames-per-second (FPS) rate, which is critical for real-time alerting. Low latency minimizes the time between a potential drowning event and its detection, a factor crucial for the effectiveness of any rescue system, as noted in [27].

Edge Deployment: The model’s lightweight nature makes it a prime candidate for deployment on edge devices. This offers advantages in remote or outdoor aquatic environments where bandwidth is limited and privacy is a concern, as video data can be processed locally without being transmitted to the cloud. The reduced dependency on high-end GPUs also lowers the overall system cost and power consumption, enhancing its practicality for widespread adoption.

Therefore, YOLO11-LiB is not merely an academic exercise in accuracy improvement but a model designed with tangible deployment scenarios in mind, addressing the critical challenges of scalability, real-time performance, and cost-effectiveness.

## 6. Conclusions

This paper proposes the YOLO11-LiB model, an improved version based on YOLO11n, to address the practical requirements of drowning detection in swimming pool scenarios. Through lightweight design, attention mechanism optimization, and multi-scale feature fusion, it effectively resolves bottlenecks in existing technologies regarding edge deployment efficiency, robustness in complex environments, and multi-scale target detection.

The main research conclusions are as follows:

- Lightweight design significantly enhances deployment adaptability: LGCBlock through synergistic optimization of LAE downsampling and GhostC3k2 structure, reduces downsampling computation by 87.5% and feature extraction overhead by 50% while preserving the integrity of key features. This substantially reduces model parameters and size, meeting deployment requirements in resource-constrained scenarios such as pool-embedded terminals.
- Attention mechanism strengthens robustness in complex environments: C2PSAiSCSA enhances perception of subtle drowning postures by combining spatial-channel decoupled attention with local context modeling. It effectively suppresses interference from water surface glare and crowd occlusion, increasing drowning recall rate (DR) to 89.7%.
- Fusion improves multi-scale detection accuracy: BiFF-Net optimizes semantic fusion of multi-scale features through its bidirectional feedback mechanism of dynamic weighting and frequency-domain enhancement. It particularly improves detection accuracy for small targets, achieving a DmAP50 of 94.1%, outperforming all comparison models.

Comprehensive performance balance validates solution effectiveness: Comparative and ablation experiments demonstrate that YOLO11-LiB achieves a DmAP50 of 94.1%, representing a 2.3% improvement over the baseline YOLO11n, while reducing model size by 19.2%. This achieves an ideal “accuracy–efficiency” balance, providing reliable technical support for real-time drowning detection.

Current research on aquatic object detection utilizing YOLO architectures demonstrates notable limitations: The visual datasets commonly employed frequently lack comprehensive coverage of complex, variable real-world aquatic environments, including adverse weather, turbid water, and low-light conditions, while exhibiting inadequate robustness under extreme optical circumstances. More critically, as single-frame detectors, YOLO-based systems fundamentally cannot capture temporal features inherent in continuous dynamic processes such as drowning, substantially elevating risks of false alarms and missed detections.

Future work must urgently prioritize constructing diversified datasets encompassing more complex and extreme scenarios, alongside exploring integration of multimodal sensory information to enhance perceptual reliability in challenging environments. Concurrently, transcending the single-frame detection paradigm through incorporation of temporal modeling or object tracking techniques is essential to analyze targets’ continuous motion states and behavioral patterns. This paradigm shift will enable more precise identification of progressive events like drowning. Collectively, these advancements will significantly improve system practicality and generalization capability, facilitating deployment of aquatic safety monitoring technologies across broader, more demanding real-world applications.

## Figures and Tables

**Table 1:** Model parameter settings.

Parameter	Value
Epoch	300
Batch Size	16
Image Size	640
Learning Rate	0.01
Optimizer	SGD
EarlyStop	100

All experiments used the same parameter settings.

**Table 2:** Comparative experimental results.

Model	DP (%)	DR (%)	DmAP50 (%)	SmAP50 (%)	FLOPs (G)	FPS	Params (M)	Size (MB)
YOLO11n	85.7	88.8	91.8	82.4	6.3	189.5	2.58	5.26
YOLO11s	88.0	89.7	91.8	84.6	21.3	182.9	9.41	18.3
YOLO11m	88.1	89.4	92.5	84.6	67.7	146.7	20.03	40.5
YOLO11l	85.9	90.9	91.2	86.1	86.6	98.3	25.28	51.2
YOLOv5	86.0	89.7	92.9	84.8	7.1	180.0	2.50	5.07
YOLOv8n	87.6	88.6	92.5	84.5	8.1	**250.9**	3.01	5.98
YOLOv9t	85.2	87.1	91.9	84.4	7.6	97.9	**1.97**	4.46
YOLOv10n	88.8	89.0	91.8	83.9	8.2	164.2	2.70	5.53
YOLO12n	80.8	83.3	90.0	81.2	6.3	126.2	2.56	5.31
RTDERT-I	**90.7**	87.8	90.0	82.3	103.4	52.8	31.99	63
Faster R-CNN	86.2	**91.5**	92.8	83.1	369.2	18.2	136.5	278
ConvNeXt	88.5	89.3	93.2	85.2	95.7	42.5	48.9	98
ViTDet	90.2	90.1	**94.5**	**86.3**	1024.0	9.8	150.2	305
RetinaNet	84.9	88.5	91.0	82.0	210.5	28.1	36.8	74.5
Ours	88.4	89.7	94.1	85.6	**6.2**	80.5	2.02	**4.25**

All experiments used the same parameter settings; The bold markings in the table are used to highlight the model that has the most outstanding performance under the corresponding evaluation metric.

**Table 3:** Ablation study results.

Model	DP (%)	DR (%)	DmAP50 (%)	SmAP50 (%)	FLOPs (G)	FPS	Params (M)	Size (MB)
YOLO11n	85.7	88.8	91.8	82.4	6.3	**189.5**	2.58	5.26
+A	86.6	87.9	92	83.8	5.9	118.8	2.21	4.58
+B	**89.7**	89.8	93.0	**85.8**	6.3	165.7	2.55	5.21
+C	84.9	**90.1**	91.7	85.4	6.8	108.6	2.43	4.99
+A+B	87.4	89.7	92.3	83.9	**5.9**	109.4	2.18	4.53
+A+B+C	88.4	89.7	**94.1**	85.6	6.2	80.5	**2.02**	**4.25**

All experiments used the same parameter settings; A: LGCBlock, B: C2PSAiSCSA, C: BiFF-Net; The bold markings in the table are used to highlight the model that has the most outstanding performance under the corresponding evaluation metric.

## Data Availability

The data and codes presented in this study are publicly available on GitHub: https://github.com/Mibugi/Drowning-detection (accessed on 3 September 2025).
